# Pressor Response to Noradrenaline in the Setting of Septic Shock: Anything New under the Sun—Dexmedetomidine, Clonidine? A Minireview

**DOI:** 10.1155/2015/863715

**Published:** 2015-12-13

**Authors:** A. Géloën, C. Pichot, S. Leroy, C. Julien, M. Ghignone, C. N. May, L. Quintin

**Affiliations:** ^1^CARMeN, INSERM U 1060, Insa de Lyon, Villeurbanne, France; ^2^Physiology (EA 4612: “Neurocardiology”), University of Lyon, 69622 Lyon, France; ^3^UMI 233, Institut de Recherche du Developpement, Montpellier, France; ^4^Florey Institute of Neuroscience and Mental Health, Parkville, VIC 3052, Australia

## Abstract

Progress over the last 50 years has led to a decline in mortality from ≈70% to ≈20% in the best series of patients with septic shock. Nevertheless, refractory septic shock still carries a mortality close to 100%. In the best series, the mortality appears related to multiple organ failure linked to comorbidities and/or an intense inflammatory response: shortening the period that the subject is exposed to circulatory instability may further lower mortality. Treatment aims at reestablishing circulation within a “central” compartment (i.e., brain, heart, and lung) but fails to reestablish a disorganized microcirculation or an adequate response to noradrenaline, the most widely used vasopressor. Indeed, steroids, nitric oxide synthase inhibitors, or donors have not achieved overwhelming acceptance in the setting of septic shock. *Counterintuitively*, *α*_2_-adrenoceptor agonists were shown to reduce noradrenaline requirements in two cases of human septic shock. This has been replicated in rat and sheep models of sepsis. In addition, some data show that *α*_2_-adrenoceptor agonists lead to an improvement in the microcirculation. Evidence-based documentation of the effects of alpha-2 agonists is needed in the setting of human septic shock.

## 1. Introduction

Following immediate resuscitation [1], the clinician treating septic shock faces* different* issues including (a) recoupling the peripheral compartment (i.e., the microcirculation) to the “central” compartment (i.e., brain, heart, and lung) and (b) restoring the pressor response to vasopressors, usually noradrenaline (NA). This minireview addresses these issues in the setting of septic shock, given the surge in interest pertaining to the use of *α*_2_-adrenoceptor agonists in this setting [2, 3].

## 2. Septic Shock

### 2.1. Septic Shock

The definition of septic shock includes a systolic blood pressure (SBP) <90 mmHg, after adequate fluid replacement (commonly >30 mL·kg-1 in <6 h) and the need for vasopressor drugs for more than 1 h [4] or for 4 h (minimal requirements of NA >0.05 *μ*g·kg-1·min-1). Earlier series have reported a death toll of ≈70% [5] and recent series still report a high mortality (27% [6], 20% [7], and 16% [8]). Refractory septic shock is defined as a requirement for NA >0.25 *μ*g·kg-1·min-1 (>1 mg·h-1/70 kg) [9] or >0.5 *μ*g·kg-1·min-1 [10]. Other definitions are (a) worsening circulatory failure despite aggressive use of vasopressors and (b) increasing lactic acidosis despite 6 h of extrarenal replacement therapy (ERRT) [11]. In a study of 51 consecutive patients with septic shock [12], an overall 45% mortality was observed. Sixteen patients presented with refractory septic shock and death (31% of the enrolled patients). The definition of refractory septic shock of this group [12] was no reversal of shock (i.e., an inability to sustain SBP >90 mmHg for >24 h without NA):

- In the refractory septic shock group, the mortality over 48 h was 19%, given the whole 51 patients: ten patients (62% of the patients in refractory septic shock) died within 48 h of circulatory failure. The mortality over 28 d (early circulatory failure and late multiple organ failure) in the refractory septic shock patients was 100%. The NA requirement was ≈2.6 *μ*g-1·kg-1·min-1, that is, ≈11 mg·h-1.
- In nonrefractory septic shock, the mortality over 28 d was 20%. The NA requirements were ≈1 *μ*g-1·kg-1·min-1, that is, ≈4 mg·h-1 [12].

### 2.2. Treatment

The initial treatment for septic shock is volume loading, but the adequacy of volume loading is poorly defined [1]. Presumably, the best index is the collapsibility of the vena cava (superior vena cava [13] or inferior vena cava) or absence of response to passive leg rising. Thus, adequacy of volume load is assessed when little or no change occurs in the diameter of the inferior or superior vena cava or when additional volume loading evokes no additional increase in cardiac output (CO). There is ongoing controversy regarding the balance between the necessity to achieve adequate volemia, during the first 24–72 h, and the necessity to avoid increased lung water by normalizing the net weight gain, as early as possible.

The second line of therapy is the use of vasopressors, usually NA, to achieve a MAP ≥65 mmHg. The dose of NA required varies from ≈1 *μ*g-1·kg-1·min-1 to ≈2.6 *μ*g-1·kg-1·min-1, respectively, in nonrefractory versus refractory septic shock (4 to 11 mg·h-1) [12]. However, the same established group [14] uses NA as high as 50–100 mg·h-1 to treat refractory septic shock. Secondly, setting the MAP ≥65 mmHg may be arbitrary: BP is too low when dealing with patients with preexisting hypertension [15] or with low functional capillary density [16]. Conversely, BP is too high if MAP is the only parameter to be followed (i.e., disregarding the indices of global tissue perfusion such as trends in arterial lactate concentration, mixed venous O_2_ saturation or superior vena cava O_2_ saturation, and arterial-venous CO_2_ gradient). There is also a controversy regarding the time NA treatment is instituted. Most commonly, NA is administered early in sepsis, especially if diastolic BP is low, but early institution of vasopressor treatment before achieving adequate global perfusion is associated with worse outcome [17] suggesting that maldistribution of blood flow may be increased by the liberal use of vasopressor in the setting of septic shock [17]. Therefore, a three-step strategy has been proposed [18]. First, as soon as possible [19], restore volume and peripheral perfusion using iterative monitoring of global tissue perfusion. Second, administer NA to maintain MAP ≥50 mmHg (lower limit of cerebral/coronary autoregulation in normal humans, with a higher MAP if coronary/cerebral perfusion is endangered). Third, optimize kidney perfusion (as an index of single organ perfusion),* after* adequate global perfusion, by increasing the dose of NA [18].

## 3. Microcirculation

### 3.1. Uncoupling between the Peripheral and Central Compartments

One of the key problems faced by the intensivist in the setting of septic shock is an “uncoupling” between the macrocirculation (“central compartment”: brain, heart, and lung) and the microcirculation. Within this schema, central compartment versus microcirculation, the kidney presents with peculiarities: given the large volume of blood it receives per time unit, the kidney is part of the central circulation. On the other hand, the microcirculation of the kidney is disrupted by sepsis, as any other major central organ: the clinical answer lies in a urine output >0.5 mL·h-1 as an index of adequate microcirculation.

Tissue blood flow is driven by metabolic demand, not by blood pressure (BP). At rest, in the healthy volunteer, this implies that the capillaries are alternatively perfused and then not perfused. In turn, this implies, in the healthy volunteer, that the blood volume needed to operate the whole circulatory system is kept to a minimum because the active part of the circulatory system is also kept to a minimum. By contrast, during exercise, muscle blood flow, under sympathetic restraint, increases 100-fold [20] with maximal capillary perfusion attained in 15 s, compatible with a metabolic demand, restrained by sympathetic activation. Diving mammals are able to store massive loads of lactate at the periphery during diving and recirculate this acid load very quickly, getting ready for the next dive within minutes [21]. Accordingly, elite long-distance runners handle severe lactic acidosis and recirculate this load quickly upon completion of run.

At variance with data gathered in the 1930s, recent results argue against the existence of precapillary sphincters that would allow independent, active control of individual capillaries. Arterioles are enmeshed in a rich plexus of sympathetic nerves and electrical stimulation leads to vasoconstriction spreading along the whole arteriole [22]. The sympathetic nervous system is activated by pressure (cardiac and vasomotor sympathetic baroreflexes), CO_2_-H^+^-O_2_ (chemoreflexes), or metabolism (metaboreflex). The large proximal arteries are controlled primarily by stimulation of *α*_1_-adrenoceptors, whereas small distal arteries are controlled mainly by stimulation of *α*_2_-adrenoceptors [20]. There is evidence that constriction of microvessels mediated by *α*_2_-adrenoceptors may be more sensitive to acidosis, compared with those mediated by *α*_1_-adrenoceptors, but it is unclear if this leads to a better local control of terminal arterioles by metabolic demand [23]. Furthermore, it is unclear how this acidosis-evoked vasodilatation of small arterioles relates to the microcirculatory dysfunction observed during septic shock and massive sympathoactivation. The untested implication is that prolonged tissue hypoxia, or prolonged unloading of arterial baroreceptors, leads to prolonged, metabolically mediated, sympathetic activation. In turn, is this sympathetic activation instrumental in perpetuating tissue hypoxia? Conversely, does sympathetic deactivation alleviate peripheral shunting?

What happens to microvascular flow in to septic shock? This is not crystal clear. However, there is evidence that, in skeletal muscle, there is a large heterogeneity in the flow rate in capillaries [24]. Given normal BP in a rat model of peritonitis (caecal ligation and perforation) [24], a decrease in continuous blood flow and of normal blood flow was observed, while an increase in stopped flow was observed. The proportion of fast to normal flow increased, possibly due to a convective arterial-venous shunt. The oxygen saturation is lower at the venular end of the capillaries. The increase in oxygen extraction (O_2_ER) was directly related to the extent of stopped flow (5 times the O_2_ER observed in controls). This corresponded to a* loss of 50% of perfused capillaries*. Taken together, these data indicate a patchy and disperse maldistribution of O_2_ during sepsis, as opposed to an inability to utilize O_2_ [24], that is, a cytopathic hypoxia [25]. The authors conclude the following: (a)* increasing the delivery of oxygen to supranormal levels may not improve tissue oxygenation if the increased O*_*2*_ *supply cannot be properly distributed and* (b)* early treatment aimed at restoring uniform distribution of O*_*2*_ *…may lead to improve outcomes* [24]. This summarizes the present challenge. The speculation is that some capillaries are vasodilated due to NO excess and thus need NO inhibition. By contrast, flow is stopped in a large proportion of the capillaries: do these stopped capillaries need NO donors? All together this makes the systemic administration of NO inhibitors versus donors a challenge.

In septic humans, a reduced density of perfused sublingual capillaries is observed in nonsurvivors [26], irrespective of a similar circulatory and oxygenation profile observed in survivors versus nonsurvivors. Survival is associated with the increase in small vessel perfusion over the first 24 h but not associated with the overall circulatory and oxygenation variables [27]. Furthermore, there is a strong association between the delay in beginning therapy and outcome, compatible with extensive microcirculatory defects and their consequences, that is, multiple organ failure [19]. Volume load improves microcirculation during early but not late sepsis [28], suggestive of damage to the microcirculation. Additionally, the first bolus of volume loading improves all the indices of microcirculation, with no further improvement with a second bolus [29]: does this imply minimizing volume load during septic shock based on a microcirculatory index? The proportion of perfused vessels is unrelated to the administration of vasopressors [26]. A weak but significant correlation exists between small vessel perfusion, increasing pH and decreasing arterial lactate levels [26], which does not necessarily imply causality. When NA was used to increase BP from 65 to 85 mmHg,* the largest increase in perfused capillary density was observed in patients presenting with the lowest perfused capillary density*, suggestive of a possible effect of BP on functional capillary density: do the sicker patients need a higher BP? By contrast, the patients with the highest baseline perfused capillary density showed a reduction in perfused capillary density [16]. This suggests that individualized titration of NA based on the state of the microcirculation may be beneficial.

Finally, no correlation was observed between the slope of recovery to thenar muscle ischemia and NA requirements [30], although a weak correlation was observed between NA requirement and recovery during ischemia of the thenar muscle [12]. Therefore, NA requirement and the extent of microcirculatory defects are poorly related: “*the alterations in thenar O*_*2*_ *saturation…are more related to the sepsis…itself and its severity than to mean arterial pressure and the dose of vasopressor agents*” [30].

## 4. Pressor Response to Noradrenaline

Reduced pressor responsive to NA is a major challenge for clinicians treating septic patients. The effects of a number of treatments have been studied to determine if they improve the reduced pressor responsiveness to NA in sepsis.

### 4.1. Nitric Oxide Inhibitors

Based on the assumption of generalized nitric oxide (NO) excess in sepsis and subsequent excessive vasodilation, NO inhibitors have been tested [31]. Briefly (a) in septic patients, NO synthase (NOS) inhibitors (N-monomethyl-L-arginine: L NNMA) increased BP and lowered CO in a dose-dependent manner [32, 33], with a 40% reduction in NA requirements [34], and (b) the changes evoked by L NNMA (inhibition of NO synthase) were reversed by L arginine [33]. However, a large study was stopped because of increased mortality in the group treated with a NOS inhibitor [35]. Studies in an ovine model of hyperdynamic septic shock showed that nonselective NOS inhibition restored BP, but not renal function, and a selective inhibitor of inducible NOS had no effect on BP or renal function [36, 37]. Therefore, NOS inhibitors do not restore, in septic shock, the delicate tuning between active, perfused capillaries and inactive, unperfused capillaries governed by local metabolic demand in the resting healthy volunteer.

Another NO inhibitor, methylene blue (MB), improved the circulatory profile (increased stroke volume and reduced tachycardia) and reduced the NA requirements by 87%, as early as one hour after beginning of administration [38, 39]. A meta-analysis favored the use of MB in hypotensive patients, including septic shock patients (mortality: MB: 16%; control: 23%) [40]. To our knowledge, no further large-scale randomized study has taken up the issue.

### 4.2. NO Donors

In a nonrandomized study, the NO donor nitroglycerin (NTG) was administered during septic shock (bolus: 0.5 mg; continuous administration: 0.5–4.0 mg·h-1), after volume load (central venous pressure >12 mmHg) and vasopressor administration. A major improvement in the microcirculation was observed, with survival in 7 out of 8 patients [41]. A similar response has also been observed with a NTG patch (12–18 mg every 4 h) [42] and in a randomized study there was evidence that NTG improved perfusion of small vessels in septic patients [43]. Furthermore, the trend in lactate concentrations improved in the NTG group. The study was not conclusive regarding whether NTG reduced the length of stay in the critical care unit, but there was a higher mortality in the NTG group [43]. However, the small size sample and the inclusion of septic patients together with septic shock patients does not allow one to reach a definitive conclusion.

### 4.3. Hydrocortisone (HSHC)

Low dose steroids generated little increase in BP in septic shock patients receiving or not receiving phenylephrine, except when very high doses of phenylephrine were used [44]. HSHC hastened the reversal of septic shock (HSHC: 3.3 days versus placebo; 5.8 days in patients in whom shock was reversed; 76 and 70% in the HSHC and placebo groups, resp.), irrespective of a positive or negative response to corticotropin. However, mortality was unchanged, irrespective of group. A higher incidence of new episodes of sepsis or septic shock was observed in the HSHC group.

### 4.4. *α*_2_-Adrenoceptor Agonists

As the drugs cited above were not overwhelmingly successful in treating sepsis, our group has examined a novel and* counterintuitive* approach: the use of *α*_2_-adrenoceptor agonists. In two cases [45], treatment with the *α*_2_-adrenoceptor agonist, clonidine (1 *μ*g·kg-1·h-1), in addition to state-of-the-art treatment, reduced NA requirements in (a) a patient presenting with HIV and terminal pulmonary sepsis (−45%) [45] and (b) a neonate presenting with necrotizing enterocolitis (−90%, submitted). In addition, we have documented this reduction in requirement for NA in rat [46] and sheep [47] experimental models of sepsis, using high and low doses, respectively, of the *α*_2_-adrenoceptor agonists, clonidine and dexmedetomidine. Furthermore, the pressor responsiveness to a noncatecholaminergic vasopressor, angiotensin II, was also reduced by clonidine treatment [47].

One possible mechanism [48] for this effect of *α*_2_-adrenoceptor agonists in sepsis is that, during septic shock, as during exercise [49], there is increased sympathetic nerve activity and endogenous plasma catecholamines [50–52] with a downregulation in responsiveness to stimulation of *α*_1_- and *β*-adrenoceptors, which may result from reduced binding or reduced sensitivity/intracellular coupling. Conversely, the other side of this working hypothesis [48] is that, during rest after exercise, or after lowering plasma catecholamine concentrations with either pharmacologically evoked *α*_2_-adrenoceptor agonists or those occurring spontaneously during recovery from sepsis, the downregulation of *α*_1_-adrenoceptors is converted to upregulation, with an increased pressor response to vasopressors.

Clonidine reduces sympathetic nerve activity to the heart and vasculature by a direct central action, which is its main mechanism of action as an antihypertensive drug [53, 54]. How can this central action of clonidine to reduce BP in hypertensive patients be reconciled with an increased pressor response and lowered NA requirement in patients with sepsis? A recent experimental study indicates that treatment with clonidine reduced renal sympathetic nerve activity from high to normal levels [47]. Together with reductions in sympathetic nerve activity to other organs, this is likely associated with a decrease in plasma catecholamines concentrations and is compatible with our working hypothesis. It remains to be determined whether the maldistribution of capillary perfusion in sepsis [24] is improved by treatment with *α*_2_-adrenoceptor agonists and if so whether this is due to its central sympathetic deactivation or to a direct vascular action.

Given our reports that clonidine reduced the requirement for NA in sepsis [45] and our demonstrations of improved pressor responsiveness in small [46] and large [47] animal models of sepsis, it is essential that evidence-based documentation of the effects of *α*_2_-adrenoceptor agonists in human septic shock is obtained. A concern may be the possible harm to the patient by using an antihypertensive agent during septic shock, indeed a bold and* counterintuitive* move. The answer appears three-fold. First, adequate volume loading before administration of the *α*_2_-adrenoceptor agonist is needed. As the microcirculation corrects slowly (as shown by the changes in arterial lactate, central O_2_ saturation, and arterial to venous CO_2_ gradient) the most expeditious way would be to optimize the central compartment: little or no collapsibility of the inferior or superior vena cava during ventilation would guarantee no more increase in CO or little response to passive leg rising. Second, the definition of an adequate BP is needed: permissive hypotension [18] (MAP ≥ 50 mmHg) versus standard MAP ≥65 mmHg [1] versus higher MAP in selected patients [15, 16]. Third, given the very high circulatory-related mortality in refractory septic shock [12], the patients in this category may be administered with a “compassionate” treatment under the Helsinki Declaration (“*where proven prophylactic, diagnostic, and therapeutic methods do not exist or have been ineffective, the physician, with informed consent from the patient, must be free to use unproven or new prophylactic, diagnostic, and therapeutic measures, if in the physician's judgment it offers hope of saving life, reestablishing health, or alleviating suffering; where possible, these measures should be made the object of research, designed to evaluate their safety and efficacy*”). The primary end-point will be increased pressor responsiveness to vasopressors. Such a clinical trial should also address whether there are improvements in the microcirculation. For, example, does sympathetic deactivation with an *α*_2_-adrenoceptor agonist reverse the peripheral microcirculatory shut-down and reduce inflammation and multiple organ failure? An end-point on mortality would require a large sample, not compatible with a preliminary trial.

### 4.5. Clonidine versus Dexmedetomidine

Which alpha-2 agonist is to be selected to head into a preliminary clinical trial? Clonidine has 2 disadvantages and one advantage: (a) a slow onset (3–6 h) when administered slowly and intravenously to evoke no precipitous sympathetic deactivation. In the context of inadequate volemia (§ treatment), precipitous sympathodeactivation will lead to a precipitous fall in BP.* A rigorous proviso should be made to address the issue of an optimized volemia before the initiation of sympathetic deactivation*. When opposed to clonidine, dexmedetomidine will be the drug of choice as its sedative effect is observed after 30–60 min. However, the issue in the setting of septic shock is not to observe a fast onset for sedation, but to observe a putative, increased pressor response to NA, without precipitous fall in BP. As the patient presenting with septic shock is to stay in the CCU for an extended period of time, a faster onset of sedative versus pressor effect will make little pharmacoeconomic difference. Our observations [45] show that clonidine increases the pressor response to NA within 2-3 h. Nevertheless, a comparison will be needed to address the superiority of any of the two clinically available alpha-2 agonists (b) in healthy volunteers, a long elimination half-life of clonidine (circa 24 h) [55] as opposed to a short elimination half-life for dexmedetomidine (circa 3 h). Any untoward effect will presumably last longer with clonidine. This is not the issue: the point is how to get increased pressor response to NA without inducing a major fall in BP during the* initial* administration of the alpha-2 agonist, and not the possible length of time of such an exaggerated fall. The answer rests with adequate volemia* before* heading to sympathetic deactivation (§ treatment). (c) Clonidine is eliminated via the kidney as opposed to dexmedetomidine eliminated via the liver. Many patients presenting with septic shock require ERRT. Thus, any overdose of clonidine will be easily eliminated. By contrast, dexmedetomidine may not generate an overdose secondary to kidney failure, easing the management. However, administration of dexmedetomidine may become tricky if the patient presents liver failure, as extra-liver replacement therapy is no widely available. Lastly, the key point is the dose of alpha-2 agonist needed to generate sympathetic deactivation, thus increased pressor response: the dose of clonidine we used [45] (1 mcg·kg-1·h-1) needs to be refined to achieve maximal sympathodeactivation with minimal side effects.

## 5. Conclusion

In healthy volunteers, the microcirculation is constantly shunting blood away from inactive to active territories and vice versa. This fine tuning allows the whole body to be adequately perfused with a blood volume of only 5 L, even in the setting of strenuous exercise. By contrast, in the setting of septic shock, the human organism apparently needs a higher blood volume (or at least reestablishment of adequate blood volume) and a recoupling of the microcirculation with the central compartment. At present, physicians are unable to emulate what humans achieve after long-distance running or diving mammals when they reach the surface, that is, reorganizing a shut-down microcirculation to force O_2_ through capillaries and generate a quick wash-out of anaerobic metabolites.

A* different* issue is the pressor response to NA, which defines, when completely blunted, refractory septic shock. Steroids increase the response to phenylephrine, but only when very high doses of phenylephrine are used [44]. NO inhibitors have been withdrawn from trial based on side-effects, possibly related to the dose of drug. Methylene blue has not been assessed in a large double blind trial to handle refractory hypotension in the setting of septic shock. In our studies of *α*_2_-adrenoceptor agonists, we have observed a large (45–90%) reduction in NA requirements in terminal septic shock [45] and in necrotizing enterocolitis. Simultaneously, in our patients [45], peripheral mottling vanished over hours: this suggests that the microcirculation may have been progressively recoupled to the central compartment. We replicated an increase in pressor responsiveness to NA with dexmedetomidine and clonidine in the setting of mild sepsis in rat [46] and sheep [47]. The working hypothesis [45, 48] is that *α*_2_-adrenoceptor agonist mediated sympathetic deactivation lowers the release of endogenous NA, allowing upregulation of vascular *α*_1_-adrenoceptors back towards normal levels. This hypothesis [45, 48] is to be put to the acid test in the setting of human septic shock, preferably refractory. Again,* a rigorous proviso should be made to address the issue of an optimized volemia before the initiation of sympathetic deactivation.*

## Abbreviations

BP: Blood pressure
CO: Cardiac output
ERRT: Extrarenal replacement therapy
HSHC: Hemisuccinate of hydrocortisone
MAP: Mean arterial pressure
MB: Methylene blue
NA: Noradrenaline
NO: Nitric oxide
NOS: NO synthase
NTG: Nitroglycerin
O2ER: Oxygen extraction ratio
SBP: Systolic blood pressure.
